# Population-based screening to detect benzodiazepine drug-drug-drug interaction signals associated with unintentional traumatic injury

**DOI:** 10.1038/s41598-022-19551-4

**Published:** 2022-09-16

**Authors:** Cheng Chen, Sean Hennessy, Colleen M. Brensinger, Emily K. Acton, Warren B. Bilker, Sophie P. Chung, Ghadeer K. Dawwas, John R. Horn, Todd A. Miano, Thanh Phuong Pham Nguyen, Charles E. Leonard

**Affiliations:** 1 Center for Real-World Effectiveness and Safety of Therapeutics, Center for Clinical Epidemiology and Biostatistics, University of Pennsylvania, Philadelphia, PA USA; 2 Department of Biostatistics, Epidemiology, and Informatics, Perelman School of Medicine, University of Pennsylvania, Philadelphia, PA USA; 3 Leonard Davis Institute of Health Economics, University of Pennsylvania, Philadelphia, PA USA; 4 Department of Systems Pharmacology and Translational Therapeutics, Perelman School of Medicine, University of Pennsylvania, Philadelphia, PA USA; 5 Translational Center of Excellence for Neuroepidemiology and Neurology Outcomes Research, Department of Neurology, Perelman School of Medicine, University of Pennsylvania, Philadelphia, PA USA; 6 Department of Psychiatry, Perelman School of Medicine, University of Pennsylvania, Philadelphia, PA USA; 7 AthenaHealth, Inc., Watertown, MA USA; 8 Department of Pharmacy, School of Pharmacy, University of Washington, Seattle, WA USA; 9 Department of Neurology, Perelman School of Medicine, University of Pennsylvania, Philadelphia, PA USA

**Keywords:** Therapeutics, Adverse effects, Drug therapy

## Abstract

Drug interactions involving benzodiazepines and related drugs (BZDs) are increasingly recognized as a contributor to increased risk of unintentional traumatic injury. Yet, it remains unknown to what extent drug interaction triads (3DIs) may amplify BZDs’ inherent injury risk. We identified BZD 3DI signals associated with increased injury rates by conducting high-throughput pharmacoepidemiologic screening of 2000–2019 Optum’s health insurance data. Using self-controlled case series design, we included patients aged ≥ 16 years with an injury while using a BZD + co-dispensed medication (i.e., base pair). During base pair-exposed observation time, we identified other co-dispensed medications as candidate interacting precipitants. Within each patient, we compared injury rates during time exposed to the drug triad versus to the base pair only using conditional Poisson regression, adjusting for time-varying covariates. We calculated rate ratios (RRs) with 95% confidence intervals (CIs) and accounted for multiple estimation via semi-Bayes shrinkage. Among the 65,123 BZD triads examined, 79 (0.1%) were associated with increased injury rates and considered 3DI signals. Adjusted RRs for signals ranged from 3.01 (95% CI = 1.53–5.94) for clonazepam + atorvastatin with cefuroxime to 1.42 (95% CI = 1.00–2.02, p = 0.049) for alprazolam + hydrocodone with tizanidine. These signals may help researchers prioritize future etiologic studies to investigate higher-order BZD interactions.

## Introduction

Benzodiazepines and related drugs (hereafter, BZDs) are increasingly prescribed to Americans to treat insomnia and anxiety^1,2^. The rate of outpatient visits that involved benzodiazepine prescribing more than doubled from 3.8% in 2003 to 7.4% in 2015^3^. Between 2015–2016, 12.6% of United States (US) adults reported using prescription benzodiazepines annually^4^. Prevalence of past-month use of nonbenzodiazepine Z-drugs (i.e., eszopiclone, zaleplon, zolpidem) among US adults also quadrupled from 0.4% in 1999–2000 to 1.6% in 2013–2014^5^. The increase in BZD use was accompanied by growth in related adverse events^4^, including traumatic injury^6,7^. By depressing the central nervous system (CNS), BZDs may cause drowsiness, light-headiness, ataxia, and impaired driving ability, leading to injurious falls and road accidents. BZD use has been consistently implicated in various types of unintentional traumatic injury, including hip fractures and motor vehicle crashes^6,8–15^.

BZD users commonly have multiple chronic conditions^16^ and thus are at high risk for polypharmacy. In a sample of commercially insured US adults, new BZD users were taking an average of six co-medications at the time of BZD initiation^17^. This is potentially concerning since co-dispensed drugs may have pharmacokinetic and/or pharmacodynamic interactions with BZDs and exacerbate their inherent injury risks. Indeed, concomitant use of an interacting drug has been associated with > two-fold risk of injury among BZD users^9,18^. Reducing these potentially preventable adverse outcomes is important to public health, as unintentional injury is a major cause of morbidity and mortality^19^. The US Senate Special Committee on Aging and the National Highway Traffic Safety Administration have emphasized identifying drug interactions involving psychoactive drugs like BZDs as a critical strategy to prevent fall-related injury and drug-impaired driving leading to motor vehicle crashes in older adults^20,21^.

Although prior etiologic studies have examined clinical consequences of pairwise BZD interactions, such as the BZD + opioid combination^9,18^, beyond pairwise BZD interactions such as drug-drug-drug interactions (3DIs) remain understudied^18^. While some 3DIs could be postulated based on known pairwise drug interactions, the complex interplay among the drug triads in the real world may necessitate independent examination^22^. Investigating BZD 3DIs is particularly relevant given the recent concern about the combination of BZDs, opioids, and skeletal muscle relaxants (SMRs)^23^. Indeed, patients on this triple therapy were found to have 1.3–2.0 times the risk of all-cause hospitalization compared with those on the BZD-opioid or BZD-SMR dual therapy^23^, raising concerns about 3DIs among these medications.

To address this knowledge gap, we conducted population-based pharmacoepidemiologic screening to identify BZD 3DI signals associated with increased rates of unintentional traumatic injury. By generating an evidence-based list of signals, we sought to help researchers prioritize future etiologic studies aimed to investigate higher-order BZD drug interactions.

## Methods

### Data source

We conducted pharmacoepidemiologic screening of Optum’s de-identified Clinformatics® Data Mart administrative data from May 1, 2000, through June 30, 2019. The database contains person-level information on enrollment status and healthcare billing records of a large US national sample of commercially insured and Medicare Advantage beneficiaries^24^ (see details in eMethods). As this study used secondary data routinely collected in healthcare, the University of Pennsylvania’s Institutional Review Board approved the study protocol (#831486) and waived the need to obtain consents from the participants. All methods were performed in accordance with the relevant guidelines and regulations.

### Study design

**Figure 1:**
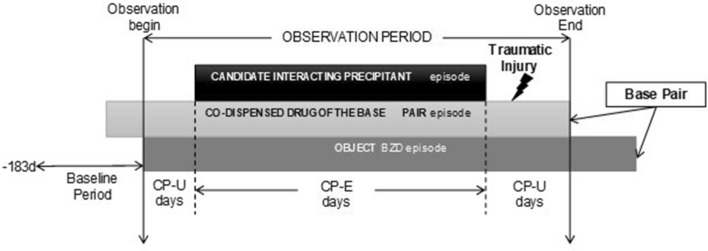
Example of benzodiazepine and related drug object + co-dispensed drug base pair exposure episode eligible for inclusion. BZD, benzodiazepine and related drug; CP-E, candidate precipitant-exposed; CP-U, candidate precipitant-unexposed.

We performed a self-controlled case series (SCCS) for each drug triad comprised of a BZD (object drug, i.e., primary affected drug of the triad), a co-dispensed drug, and a candidate interacting precipitant drug (i.e., primary affecting drug of the triad) (see Fig. 1 for an illustration of the study design). We refer to the BZD + co-dispensed drug as the base pair. Within each study individual, we compared the injury rates during time exposed to the drug triad versus to the base pair only. We selected the SCCS design since this within-person epidemiologic approach cancels out time-invariant confounders automatically^25^, is highly computationally efficient^25^, and has been used widely for drug interaction research^26–34^.

### Study samples

For each base pair, the study sample consisted of patients aged ≥ 16 years old who: (1) initiated a BZD of interest, defined as filling a prescription for BZD without filling one during the 183 days prior (i.e., baseline period); (2) were continuously enrolled during the baseline period; (3) had supply of the co-dispensed drug of the base pair while continuously using the BZD (i.e., were exposed to the base pair); (4) experienced an outcome event during the continuous exposure to the base pair; and (5) used at least one valid candidate interacting precipitant drug (see “Exposure”). BZDs under study were: (1) benzodiazepine receptor agonists, including anxiolytic benzodiazepines (alprazolam, chlordiazepoxide, clobazam, clonazepam, clorazepate, diazepam, lorazepam, oxazepam), hypnotic benzodiazepines (estazolam, flurazepam, quazepam, temazepam, triazolam), and nonbenzodiazepine Z-drugs (eszopiclone, zaleplon, zolpidem); and (2) related drugs, including dual orexin receptor antagonists (suvorexant) and melatonin receptor agonists (ramelteon, tasimelteon).

### Follow-up

Patients were followed from the first day of base pair use until: (1) discontinuation of the base pair, defined as ≥ 1 day without supply of either the BZD or co-dispensed drug; to permit late prescription fills consistent with imperfect adherence, we extended BZD and co-dispensed drug days’ supply values by 20%; (2) switching to non-solid formulation of the BZD; (3) health plan disenrollment; or (4) June 30, 2019, whichever occurred first.

### Exposure

The exposure of interest was use (versus non-use) of each candidate interacting precipitant, operationalized as any orally administered medication dispensed during the base pair-defined observation time. Using pharmacy claim dispensing dates and days’ supply, we categorized each observation day as exposed (if covered by the base pair + candidate interacting precipitant) and unexposed (if covered by the base pair only). Since co-dispensed drugs (A and B) given with BZD can be classified as either co-dispensed drug of the base pair or the candidate interacting precipitant, we examined both scenarios: (1) BZD + drug A (base pair) with drug B (candidate interacting precipitant) vs. without drug B; and (2) BZD + drug B (base pair) with drug A (candidate interacting precipitant) vs. without drug A. We used Lexicon Plus (Cerner Multum: Denver, CO, US) to assign co-dispensed drug of the base pair and the candidate interacting precipitants to pharmacologic and therapeutic classes.

### Outcome

The primary outcome of interest was unintentional traumatic injury, defined as an emergency department (ED) visit with an any-position diagnosis or an inpatient hospitalization with the principal diagnosis indicative of injury. We followed the injury definition developed by the American College of Surgeon’s National Trauma Data Bank Data Standard^35^, and further excluded burns as they are unlikely to be attributable to BZD use^36^. Secondary outcomes included: (1) hip fracture, defined as having a principal inpatient discharge diagnosis indicative of typical open and closed hip fractures, excluding pathological hip fractures and atypical hip fractures; and (2) motor vehicle crash while the individual was driving, defined as having an unintentional injury plus an external cause of injury code for unintended traffic or nontraffic accident, excluding crashes of a self-inflicted, assault, or undetermined manner^37^. All outcomes were identified by the International Classification of Diseases, Ninth Revision, Clinical Modification, or Tenth Revision (ICD-9-CM or ICD-10) diagnostic codes on the medical claims within the Optum’s de-identified Clinformatics® Data Mart administrative data. We present algorithms, diagnostic codes, and their performance metrics in eTable 1.

### Covariates

As the SCCS design inherently controls for time-invariant but not time-varying confounders^25^, we controlled for key covariates that may have changed during the observation time. We assessed the following variables on each observation day: (1) the average daily BZD dose, dichotomized based on the median; (2) follow-up month^38^, dichotomized based on two-month; and (3) ever having a prior any-position, any-claim type diagnosis of traumatic injury (yes/no)^25^.

### Statistical analysis

We used conditional Poisson regression to estimate rate ratios (RRs) and 95% confidence intervals (CIs) comparing injury rates during candidate precipitant-exposed vs. candidate precipitant-unexposed observation days, i.e., $\frac{{rate_{BZD - base \;pair\; + \;candidate\; interacting\; precipitant} }}{{rate_{BZD - base\; pair} }}$, adjusting for the covariates named above. To avoid statistical instability, we refrained from estimating RRs if: (1) a base pair-candidate interacting precipitant combination had ≤ 5 exposed patients; (2) no events occurred during candidate interacting precipitant-exposed time; (3) the conditional Poisson regression model could not converge; or 4) the variance of the beta estimate for the parameter of interest was > 10. To reduce the chance for false-positive findings due to multiple testing, we adjusted RRs using semi-Bayes shrinkage^39,40^. See additional details in eMethods.

## Results

**Figure 2:**
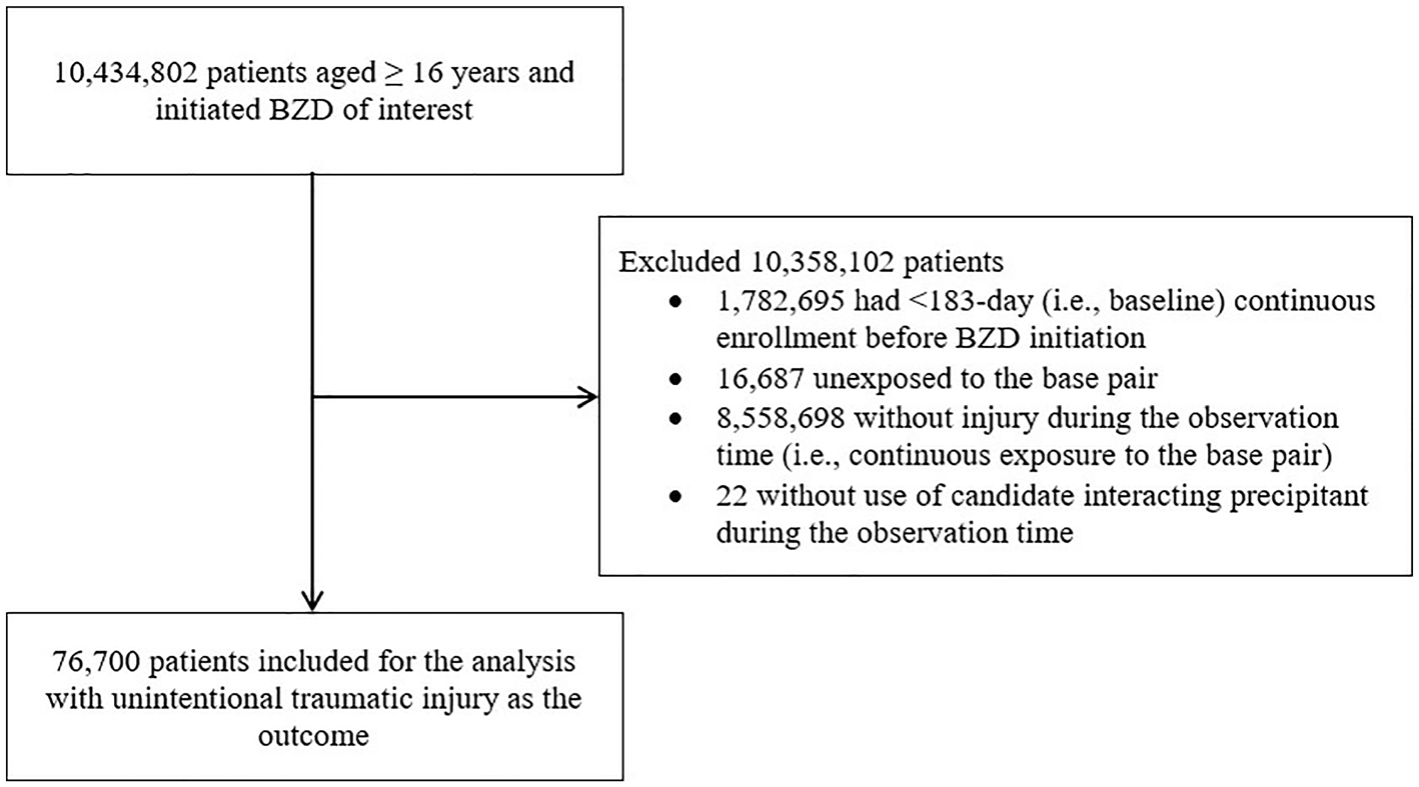
Flowchart of sample selection for the analysis for unintentional traumatic injury. BZD, benzodiazepine and related drug.

We included 76,700 BZD users in analyses of unintentional traumatic injury (see the flowchart of sample selection in Fig. 2). Table 1 summarizes patient characteristics by the BZD drug samples they contributed to. For the five most commonly used BZDs, these samples included 17,033, 16,851, 13,351, 13,004, and 9566 users of alprazolam, zolpidem, clonazepam, lorazepam, and diazepam, respectively. Most users were white (70.2–71.8%) and female (61.1–69.8%). In analyses of secondary outcomes, we included 2887 BZD users for typical hip fracture (eTable 2) and 629 BZD users for motor vehicle crash (eTable 3).

**Table 1:** Characteristics of benzodiazepine and related drug users experiencing an unintentional traumatic injury.

		Benzodiazepine and Related Drugs (N = 76,700 persons in total^a^)							
		Alprazolam	Chlordiazepoxide	Clobazam	Clonazepam	Clorazepate	Diazepam	Eszopiclone	Flurazepam
Persons	Count	17,033	939	36	13,351	440	966	2891	254
Days of observation period, per person	Median (Q1–Q3)	64.0 (37.0–229.0)	37.0 (20.0–100.0)	48.0 (32.0–343.0)	95.0 (37.0–250.0)	53.0 (37.0–150.0)	29.0 (10.0–77.0)	68.0 (37.0–191.0)	60.5 (37.0–164.5)
Days of observation	Count	3,729,726	71,806	1734	2,529,898	59,172	851,794	374,719	14,403
Age in years	Median (Q1–Q3)	64.5 (50.0–76.3)	60.2 (48.7–73.0)	49.7 (29.5–60.7)	60.6 (47.6–72.7)	68.6 (56.1–77.6)	57.9 (45.7–70.4)	58.3 (47.8–71.7)	58.4 (49.4–73.5)
Sex, count (%)	Female	11,884 (69.8)	441 (59.8)	7 (63.6)	7657 (66.5)	259 (76.4)	5061 (61.1)	1457 (65.8)	71 (68.3)
Race, count (%)	White	12,075 (70.9)	504 (68.4)	4 (36.4)	8165 (71.0)	227 (67.0)	5819 (70.2)	1680 (75.8)	71 (68.3)
	African American	1495 (8.8)	53 (7.2)	2 (18.2)	1017 (8.8)	18 (5.3)	764 (9.2)	159 (7.2)	6 (5.8)
	Hispanic	1360 (8.0)	62 (8.4)	3 (27.3)	836 (7.3)	34 (10.0)	557 (6.7)	147 (6.6)	6 (5.8)
	Asian	188 (1.1)	6 (0.8)	0 (0.0)	124 (1.1)	4 (1.2)	94 (1.1)	18 (0.8)	3 (2.9)
	Unknown	1915 (11.2)	112 (15.2)	2 (18.2)	1366 (11.9)	56 (16.5)	1054 (12.7)	211 (9.5)	18 (17.3)

		Lorazepam	Oxazepam	Ramelteon	Suvorexant	Temazepam	Triazolam	Zaleplon	Zolpidem
Persons	Count	13,004	6	550	527	4699	194	634	16,851
Days of observation period, per person	Median (Q1–Q3)	37.0 (25.0–137.0)	92.0 (25.0–347.0)	42.5 (37.0–121.0)	63.0 (37.0–165.0)	73.0 (37.0–192.0)	65.0 (37.0–194.0)	37.0 (37.0–99.0)	62.0 (37.0–137.0)
Days of observation	Count	2,025,647	1012	66,306	75,202	816,352	28,820	55,855	2,385,936
Age in years	Median (Q1-Q3)	72.0 (57.6–81.8)	74.5 (58.3–83.6)	67.2 (53.6–79.6)	69.5 (59.8–77.9)	70.1 (57.1–79.3)	63.7 (50.2–76.5)	56.9 (45.6–69.9)	66.1 (52.3–76.5)
Sex, count (%)	Female	9072 (69.8)	5 (83.3)	352 (64.0)	376 (71.3)	2992 (63.7)	135 (69.6)	427 (67.4)	10,854 (64.4)
Race, count (%)	White	9336 (71.8)	5 (83.3)	381 (69.3)	353 (67.0)	3171 (67.5)	141 (72.7)	466 (73.5)	11,904 (70.6)
	African American	1105 (8.5)	0 (0.0)	63 (11.5)	66 (12.5)	416 (8.9)	9 (4.6)	44 (6.9)	1516 (9.0)
	Hispanic	884 (6.8)	0 (0.0)	39 (7.1)	42 (8.0)	484 (10.3)	8 (4.1)	31 (4.9)	1203 (7.1)
	Asian	160 (1.2)	0 (0.0)	9 (1.6)	6 (1.1)	64 (1.4)	2 (1.0)	9 (1.4)	293 (1.7)
	Unknown	1519 (11.7)	1 (16.7)	58 (10.5)	60 (11.4)	564 (12.0)	34 (17.5)	84 (13.2)	1935 (11.5)

Q, quartile.

^a^A person may have contributed to multiple drug episodes in the analysis.

Estazolam, quazepam, and tasimelteon were examined but did not have eligible samples with statistically stable models because none of the base pair-candidate interacting precipitant combinations had ≥ 5 exposed patients.

Table 2 provides summary data on RRs for unintentional traumatic injury, before and after covariate adjustment. We examined a total of 65,123 BZD drug triads in adjusted analyses, including 13,685, 12,887, 12,339, 12,231, and 6922 for alprazolam, clonazepam, lorazepam, zolpidem, and diazepam as object drugs, respectively. A total of 79 drug triads—28 for alprazolam, 23 for clonazepam, 19 for lorazepam, 8 for zolpidem, and 1 for diazepam—had statistically elevated RRs after semi-Bayes shrinkage and were thus considered potential 3DI signals. Volcano plots in eFigure 1 graphically depict semi-Bayes shrunk adjusted RRs for these five BZDs; corresponding sensitivity analyses using an alternative variance parameter for semi-Bayes shrinkage yielded similar findings (eFigure 2). We did not observe elevated RRs for base pairs including other BZDs. eTable 4 and eTable 5 provide summary data for typical hip fracture and motor vehicle crash, respectively. We did not observe elevated RRs for any BZD for either of these secondary outcomes. Visualization of all drug triads screened and their associated RRs can be accessed via GitHub and shinyApp links provided in eResults.

**Table 2:** Summary data on rate ratios for unintentional traumatic injury, by benzodiazepine and related drug.

	Benzodiazepine and related drugs^a^							
	Alprazolam	Chlordiazepoxide	Clobazam	Clonazepam	Clorazepate	Diazepam	Eszopiclone	Flurazepam
**Unadjusted analyses**								
Number of base pair-candidate interacting precipitant triads examined	16,668	515	1	14,684	140	8206	3417	19
Range of RRs after semi-Bayes shrinkage, min to max	0.40–2.54	0.53–1.41	1.63–1.63	0.37–2.77	0.43–0.93	0.40–1.92	0.42–1.82	0.53–1.02
Number of triads with statistically significantly elevated ratio of RRs	17	0	0	21	0	0	0	0
**Confounder-adjusted analyses**								
Number of base pair-candidate interacting precipitant triads examined	13,685	154	No valid models^b^	12,887	9	6922	2262	No valid models^b^
Range of RRs after semi-Bayes shrinkage, min to max	0.38–2.48	0.64–1.50		0.33–3.01	0.46–0.58	0.37–2.10	0.42–1.89	
Number of triads with statistically significantly elevated ratio of RRs (i.e., potential 3DI signals)	28	0		23	0	1	0	

	Lorazepam	Oxazepam	Ramelteon	Suvorexant	Temazepam	Triazolam	Zaleplon	Zolpidem
**Unadjusted analyses**								
Number of base pair-candidate interacting precipitant triads examined	13,911	1	524	694	5225	34	390	14,508
Range of RRs after semi-Bayes shrinkage, min to max	0.38–2.66	6.09–6.09	0.46–1.12	0.49–1.36	0.41–2.09	0.59–0.86	0.50–1.58	0.36–2.40
Number of triads with statistically significantly elevated ratio of RRs	10	1	0	0	0	0	0	0
**Confounder-adjusted analyses**								
Number of base pair-candidate interacting precipitant triads examined	12,339	No valid models^b^	No valid models^b^	292	4300	10	32	12,231
Range of RRs after semi-Bayes shrinkage, min to max	0.39–2.54			0.60–1.44	0.39–1.91	0.95–1.36	0.59–1.26	0.38–2.49
Number of triads with statistically significantly elevated ratio of RRs (i.e., potential 3DI signals)	19			0	0	0	0	8

max, maximum; min, minimum; RR, rate ratio.

^a^Estazolam, quazepam, and tasimelteon were also examined but no models were run because none of the base pair-candidate interacting precipitant combinations had ≥ 5 exposed patients.

^b^Models were run, but none had valid results (i.e., either model did not converge or variance estimate > 10).

Table 3 lists 3DI signals (N = 79) by BZD and therapeutic category of the co-dispensed drug of the base pair. The most common co-dispensed drugs for BZD signals included CNS with CNS agents (N = 13), cardiovascular with CNS agents (N = 11), and CNS with anti-infective agents (N = 7). Statistically elevated adjusted RRs for unintentional traumatic injury after semi-Bayes shrinkage ranged from 3.01 (95% CI = 1.53–5.94) for clonazepam + atorvastatin with cefuroxime to 1.42 (95% CI = 1.00–2.02, p = 0.049) for alprazolam + hydrocodone with tizanidine.

**Table 3:** Benzodiazepine and related drugs (BZD) drug-drug-drug interaction signals of potential clinical concern given statistically significantly increased rates of unintentional traumatic injury, by commonly used BZD and therapeutic category of co-dispensed drug of the base pair.

BZD	Therapeutic class of co-dispensed drug of the base pair	Co-dispensed drug of the base pair	Candidate Interacting Precipitant	Rate ratio, semi-Bayes shrunk and adjusted^a^	95% confidence interval
ALPRAZOLAM	Cardiovascular	Fenofibrate	Ciprofloxacin	2.15	1.01–4.57
		Amlodipine	Pregabalin	2.02	1.10–3.69
		Diltiazem	Gabapentin	1.94	1.06–3.52
		Carvedilol	Gabapentin	1.69	1.04–2.75
		Lisinopril	Gabapentin	1.42	1.03–1.97
	Central nervous system	Methadone	Ondansetron	2.48	1.16–5.31
		Meloxicam	Fluconazole	2.34	1.13–4.85
		Aripiprazole	Ondansetron	2.25	1.10–4.64
		Gabapentin	Fluconazole	2.22	1.25–3.94
		Quetiapine	Levothyroxine	2.05	1.06–3.95
		Oxycodone	Morphine	1.80	1.09–2.96
		Quetiapine	Omeprazole	1.79	1.00–3.18
		Gabapentin	Quetiapine	1.72	1.02–2.91
		Oxycodone	Levothyroxine	1.72	1.06–2.77
		Hydrocodone	Sulfamethoxazole	1.60	1.02–2.50
		Hydrocodone	Trimethoprim	1.60	1.02–2.49
		Hydrocodone	Gabapentin	1.45	1.07–1.97
		Hydrocodone	Tizanidine	1.42	1.00–2.02**
	Endocrine and metabolic	Alendronate	Fluconazole	2.16	1.04–4.49
		Levothyroxine	Risperidone	1.92	1.03–3.60
		Levothyroxine	Trimethoprim	1.53	1.01–2.30
		Levothyroxine	Sulfamethoxazole	1.51	1.00–2.28
	Gastrointestinal	Ranitidine	Furosemide	2.26	1.20–4.24
		Omeprazole	Propranolol	2.12	1.02–4.41
	Hematological	Clopidogrel	Meloxicam	2.22	1.11–4.45
	Renal and genitourinary	Furosemide	Metolazone	2.00	1.02–3.91
		Furosemide	Sulfamethoxazole	1.80	1.08–3.00
		Furosemide	Trimethoprim	1.80	1.08–2.99
CLONAZEPAM	Cardiovascular	Atorvastatin	Cefuroxime	3.01	1.53–5.94
		Simvastatin	Clindamycin	2.46	1.27–4.77
		Amlodipine	Pantoprazole	2.00	1.16–3.45
		Metoprolol	Duloxetine	1.93	1.13–3.29
	Central nervous system	Meloxicam	Furosemide	2.96	1.54–5.69
		Topiramate	Sumatriptan	2.53	1.28–5.00
		Memantine	Tramadol	2.24	1.15–4.36
		Donepezil	Metoprolol	2.17	1.08–4.33
		Mirtazapine	Promethazine	2.15	1.08–4.30
		Oxycodone	Hydroxyzine	2.12	1.20–3.73
		Meloxicam	Prednisone	2.07	1.19–3.61
		Escitalopram	Metoprolol	2.00	1.06–3.78
		Sertraline	Gabapentin	1.71	1.05–2.77
		Gabapentin	Duloxetine	1.61	1.02–2.54
	Endocrine and metabolic	Metformin	Nitrofurantoin	2.04	1.04–4.00
		Metformin	Clindamycin	2.03	1.02–4.04
		Levothyroxine	Omeprazole	1.54	1.06–2.25
		Levothyroxine	Gabapentin	1.49	1.08–2.04
	Gastrointestinal	Omeprazole	Atorvastatin	1.68	1.02–2.78
		Omeprazole	Gabapentin	1.55	1.09–2.21
	Hematological	Clopidogrel	Furosemide	1.68	1.02–2.78
	Renal and genitourinary	Hydrochlorothiazide	Amlodipine	1.66	1.02–2.70
		Hydrochlorothiazide	Lisinopril	1.58	1.02–2.44
DIAZEPAM	Central nervous system	Fluoxetine	Tramadol	2.05	1.03–4.08
LORAZEPAM	Cardiovascular	Benazepril	Trimethoprim	2.27	1.08–4.75
		Benazepril	Sulfamethoxazole	2.15	1.02–4.55
		Fenofibrate	Hydrocodone	1.90	1.06–3.40
		Amlodipine	Escitalopram	1.84	1.05–3.22
		Digoxin	Hydrocodone	1.77	1.02–3.07
		Metoprolol	Escitalopram	1.75	1.04–2.97
		Amlodipine	Sulfamethoxazole	1.66	1.02–2.72
	Central nervous system	Memantine	Gabapentin	2.15	1.03–4.50
		Venlafaxine	Hydrochlorothiazide	2.06	1.07–3.98
		Escitalopram	Nitrofurantoin	1.93	1.03–3.64
		Citalopram	Sulfamethoxazole	1.87	1.07–3.28
		Citalopram	Trimethoprim	1.77	1.01–3.11
		Hydrocodone	Levothyroxine	1.74	1.05–2.90
	Endocrine and metabolic	Metformin	Escitalopram	2.01	1.02–3.94
		Levothyroxine	Atorvastatin	1.69	1.08–2.64
	Gastrointestinal	Famotidine	Ondansetron	2.54	1.24–5.24
		Omeprazole	Nitrofurantoin	2.09	1.25–3.50
	Renal and genitourinary	Furosemide	Clindamycin	2.27	1.21–4.25
		Hydrochlorothiazide	Escitalopram	1.78	1.01–3.15
ZOLPIDEM	Cardiovascular	Valsartan	Codeine	2.05	1.04–4.04
		Amlodipine	Cyclobenzaprine	2.01	1.21–3.34
		Amlodipine	Omeprazole	1.68	1.05–2.70
	Central nervous system	Amitriptyline	Pantoprazole	2.14	1.02–4.52
	Endocrine and metabolic	Levothyroxine	Meloxicam	1.63	1.01–2.61
	Hematological	Warfarin	Azithromycin	2.15	1.11–4.15
		Clopidogrel	Gabapentin	2.06	1.17–3.64
	Renal and genitourinary	Tamsulosin	Tramadol	1.98	1.14–3.43

**p = 0.049.

^a^Rate ratio was calculated as outcome rates during candidate precipitant-exposed person-time divided by outcome rates during candidate precipitant-unexposed days, i.e., $\frac{{\mathrm{rate}}_{\mathrm{BZD\,\, base \,\,pair}+\mathrm{candidate \,\,precipitant}}}{{\mathrm{rate}}_{\mathrm{BZD \,\,base\,\, pair}}}$, adjusting for the following time-varying covariates: average daily dose of BZD, follow-up month, and ever having a prior traumatic injury of interest.

## Discussion

We conducted high-throughput pharmacoepidemiologic screening of real-world data to identify BZD 3DIs that were associated with unintentional traumatic injury. Among 65,123 BZD-base pairs coupled with a candidate interacting precipitant, we identified 79 potential 3DI signals in adjusted analyses, all involving one of the five most commonly dispensed BZDs—alprazolam, clonazepam, lorazepam, zolpidem, or diazepam. We also screened, but detected no signals, for hip fracture or motor vehicle crash, prespecified subsets of unintentional traumatic injury for which we had much smaller samples.

Although drug interactions are increasingly recognized as an important modifiable risk factor for BZD-related injuries^9,18^, few studies have examined to what extent higher-order interactions may contribute to such risks. One exception is the investigation of triads comprised of a BZD with two other CNS-active agents^41^, such as the combination of BZD, opioid, and SMR^23^. The present study yielded many expected results for this combination. For example, our observation that the addition of tizanidine to alprazolam + hydrocodone was associated with 1.40-fold increased rate of injury aligns with a recent investigation showing that combining these drug classes led to a higher risk of all-cause hospitalization^23^. Moreover, 16% of our identified 3DI signals comprised of BZDs and two other CNS agents. The finding that addition of two CNS agents (vs. one CNS agent) to BZD is associated with increased rate of injury seems to corroborate a previously demonstrated relationship between the total number of CNS medications and risk of injurious falls/fractures^41^. However, because many of these interacting CNS agents may cause injuries when used alone, whether our observed 3DI signals represent synergism or additivity remains to be determined.

Several other identified 3DI signals deserve further investigation, given their relatively high RRs and biological plausibility. We categorize these signals based on putative mechanisms. First is a candidate interacting precipitant that may pharmacokinetically interact with the BZD and/or the co-dispensed drug in the base pair, thereby compounding the pharmacodynamic interaction between these two within the base pair. For example, the RR = 2.15 for promethazine with clonazepam + mirtazapine may be potentially explained by promethazine’s inhibition of mirtazapine’s metabolism via cytochrome P450 (CYP) 2D6 that has been shown in in vitro studies^42^ and the resulting enhancement of the pharmacodynamic interaction between mirtazapine and clonazepam. Second is the CNS depressing effect of the candidate interacting precipitant may worsen that resulting from a pairwise pharmacokinetic interaction between BZD and the co-dispensed drug in the base pair. For example, the RR = 1.94 for gabapentin with alprazolam + diltiazem may be possible if gabapentin augments CNS depression arising from the pharmacokinetic interaction between alprazolam (a CYP3A4 substrate)^43^ and diltiazem (a moderate CYP3A4 inhibitor)^44^. Third is orthostatic hypotensive effects of the candidate interacting precipitant that may compound CNS depression from BZDs and/or the co-dispensed drug in the base pair. Examples include hydrochlorothiazide with lorazepam + venlafaxine (RR = 2.06) and metoprolol with clonazepam + escitalopram (RR = 2.00). As the resources to examine the clinical sequelae of 3DIs are likely limited, researchers may consider the above signals as targets for future etiologic studies.

We also identified numerous 3DI signals with an anti-infective as the candidate interacting precipitant, some of which may be biologically plausible. Examples include fluconazole with alprazolam + meloxicam (RR = 2.34) and alprazolam + gabapentin (RR = 2.22), respectively. By inhibiting^45^ alprazolam’s metabolism via CYP3A4^43^, fluconazole may increase the concentration of alprazolam and enhance the pharmacodynamic interactions between alprazolam and meloxicam or gabapentin, potentially explaining the observed RRs. However, underlying mechanisms for most of the anti-infective signals remain unknown, including that with the highest RR—clonazepam + atorvastatin with cefuroxime (RR = 3.01). One possible explanation might be within-person confounding by indication, since infection may suppress drug metabolism via CYP450 pathways, elevating plasma concentrations of the BZD and/or the co-dispensed drug in the base pair (if they are metabolized via CYP450 pathways)^46^, and amplifying their inherent risks.

Our study has several strengths. First, using the healthcare records of millions of enrollees, we were able to study 3DIs that would be hard to study in smaller settings. Second, we used the analytic SCCS design, a rigorous approach that is not subject to confounding from time-invariant factors. Third, we examined signals associated with traumatic injury, a potentially preventable outcome of major public health importance. Fourth, we defined our study outcome using validated algorithms. Finally, we reduced false-positive signals and increased the specificity of our findings via semi-Bayes shrinkage.

Our study has several limitations. First, because we identified signals by comparing injury rates during person-time exposed to BZD-base pair with versus without a candidate interacting precipitant, some signals may represent inherent risk of the candidate interacting precipitant or pairwise interactions rather than true 3DIs. Future etiologic studies using negative control of each component of the drug triad may help elucidate the nature of the observed signals. Second, the pharmacy records capture only prescription fills without information on whether patients took the drug as recorded and therefore may introduce exposure misclassification, which may bias the results towards either direction. Third, our study may be susceptible to reverse causation. For example, if clinicians prescribed candidate interacting precipitants (such as anti-infectives) for early symptoms of an injury that later resulted in ED presentation or hospitalization, we may see elevated injury rates during the candidate precipitant-exposed time and consider the precipitant as interacting with the base pair. Future etiologic studies using active comparators for the candidate interacting precipitants may help address this limitation. Fourth, some unadjusted time-varying confounders, such as comorbidities, may have explained our findings. Given the high-throughput nature of our screening study, it was impractical to adjust for a comprehensive list of potential time-varying confounders. Yet, we accounted for the key covariates that were most likely to change during the observation period and explain the increased injury rates. Finally, we cannot rule out the possibility that some of our findings may be due to chance.

## Conclusions

We identified 79 potential BZD drug-drug-drug interactions with elevated rates of unintentional traumatic injury. These signals included the five most commonly used BZDs—alprazolam, clonazepam, zolpidem, lorazepam, or diazepam. Our findings may provide important targets to guide hypothesis generation and prioritize future etiological investigations into higher-order BZD interactions and risk for unintentional traumatic injury.

## Supplementary Information

Supplementary Information.

## Data Availability

The data that support the findings of this study are available from Optum’s de-identified Clinformatics® Data Mart. Restrictions apply to the availability of these data, which were used under license for this study. Data are available from the authors with the permission of Optum.
