# Supplementary material for: Population-based screening to detect benzodiazepine drug-drug-drug interaction signals associated with unintentional traumatic injury
Source: Sci Rep. 2022 Sep 16;12:15569. doi: 10.1038/s41598-022-19551-4 (PMC9481644; doi:10.1038/s41598-022-19551-4)
Supplement: Supplementary file 1 — Supplementary Information. [file 41598_2022_19551_MOESM1_ESM.docx]

**Supplementary Material**

**Title: Population-based Screening to Detect Benzodiazepine Drug-Drug-Drug Interaction Signals Associated with Unintentional Traumatic Injury**

**Journal:** Scientific Reports

**Authors:** Cheng Chen, PhD^1,2^, Sean Hennessy, PharmD, PhD^1,2,3,4^, Colleen M. Brensinger, MS^1,2^, Emily K. Acton, MSCE^1,2,5^, Warren B. Bilker, PhD^1,6^, Sophie P. Chung, PharmD^7^, Ghadeer K. Dawwas, PhD, MSc, MBA^1,2,3^, John R. Horn, PharmD^8^, Todd A. Miano, PharmD, PhD^1,2^, Thanh Phuong Pham Nguyen, PharmD, MBA, MSCE^1,5,9^, Charles E. Leonard, PharmD, MSCE^1,2,3^

**Affiliations:**

1. Center for Real-World Effectiveness and Safety of Therapeutics, Center for Clinical Epidemiology and Biostatistics, University of Pennsylvania (Philadelphia, PA, US)
2. Department of Biostatistics, Epidemiology, and Informatics, Perelman School of Medicine, University of Pennsylvania (Philadelphia, PA, US)
3. Leonard Davis Institute of Health Economics, University of Pennsylvania (Philadelphia, PA, US)
4. Department of Systems Pharmacology and Translational Therapeutics, Perelman School of Medicine, University of Pennsylvania (Philadelphia, PA, US)
5. Translational Center of Excellence for Neuroepidemiology and Neurology Outcomes Research, Department of Neurology, Perelman School of Medicine, University of Pennsylvania (Philadelphia, PA, US)
6. Department of Psychiatry, Perelman School of Medicine, University of Pennsylvania (Philadelphia, PA, US)
7. AthenaHealth, Inc. (Watertown, MA, US)
8. Department of Pharmacy, School of Pharmacy, University of Washington (Seattle, WA, US)
9. Department of Neurology, University of Pennsylvania Perelman School of Medicine, Philadelphia, PA

**Correspondence**

Charles E. Leonard, Department of Biostatistics, Epidemiology, and Informatics, Perelman School of Medicine, University of Pennsylvania, 807 Blockley Hall, 423 Guardian Drive, Philadelphia, PA 19104, US. Telephone: 1-215-573-2663. Fax: 1-215-573-5315. Email: celeonar@pennmedicine.upenn.edu.

**eMethods.** Supplemental methods

**eTable 1.** Outcome definitions

**eTable 2.** Characteristics of benzodiazepine and related drug users experiencing a typical hip fracture

**eTable 3.** Characteristics of benzodiazepine and related drug users experiencing a motor vehicle crash while individual was driving

**eTable 4.** Summary data on rate ratios for typical hip fracture, by benzodiazepine and related drug

**eTable 5.** Summary data on rate ratios for motor vehicle crash while individual was driving, by benzodiazepine and related drug

**eFigure 1.** Commonly prescribed benzodiazepine and related drugs + co-dispensed drug of the base pair with candidate interacting precipitant associations with unintentional traumatic injury, σ^2^ = 0.25.

**eFigure 2.** Commonly prescribed benzodiazepine and related drugs + co-dispensed drug of the base pair with candidate interacting precipitant associations with unintentional traumatic injury, σ^2^ = 0.67.

**eResults.** Links to visualization of all benzodiazepine and related drugs triads screened and their associated rate ratios

**eMethods.** Supplemental methods

Optum

Optum data elements include demographics (e.g., age, sex, race); enrollment periods; medical encounters (e.g., ambulatory care visits, emergency department visits, inpatient hospitalizations) and their accompanying diagnoses and procedures; pharmacy dispensings (excluding in inpatient settings); and laboratory orders and results for a subset of beneficiaries. We selected Optum as our data source because of its generalizability to the United States population, as ~65% of Americans receive healthcare coverage via commercial health plans or Medicare. Additional detail on Optum data is available at <https://www.optum.com/content/dam/optum/resources/productSheets/Clinformatics_for_Data_Mart.pdf>.

Semi-Bayes shrinkage

Operationally, we prespecified a variance (σ^2^ = 0.25) to assume that 95% of true rate ratios would be within an unspecified 7-fold range of each other (e.g., 0.5–3.5, since stronger drug interaction associations would likely have already been identified via case reports), then shrunk outlying effect estimates toward their geometric mean; this approach is relevant to **eFigure 1**. In a prespecified secondary analysis, we increased the variance (σ^2^ = 0.67) to assume that 95% of true rate ratios would be within an unspecified 25-fold range of each other; this approach is relevant to **eFigure 2**.

**eTable 1.** Outcome definitions

| **Priority** | **Outcome** | **Discharge diagnosis ICD-9-CM code(s)** | **Discharge diagnosis ICD-10-CM code(s)** | **Discharge diagnosis position and claim type** | **Performance characteristics and/or other supporting information** |
| --- | --- | --- | --- | --- | --- |
| Primary | Unintentional traumatic injury | Fracture (800-829)  Dislocation (830-839);  Sprain/strain (840-848);  Intracranial injury (850-854);  Internal injury of thorax, abdomen, or pelvis (860-869);  Open wound (870-897);  Injury to blood vessels (900-904);  Crushing injury (925-929);  Injury to nerves or spinal cord (950-957);  Certain traumatic complications and unspecified injuries (958-959) | Injuries (S00–S99 with a 7^th^ character modifier of A, B, or C only, excluding superficial injuries indicated by S00, S10, S20, S30, S40, S50, S60, S70, S80, or S90);  Unspecified multiple injuries (T07 with a 7^th^ character modifier of A only);  Injury of unspecified body region (T14 with a 7^th^ character modifier of A only, excluding T14.91* [suicide attempt);  Traumatic compartment syndrome (T79.A1–T79.A9 with 7th character modifier of A only). | Any-position discharge diagnosis on an ED claim  or  Principal inpatient discharge diagnosis on an inpatient hospitalization claim | Adapted from the injury definition used by the American College of Surgeons’ NTDB Data Standard (version 03.2015 for ICD-9-CM codes; version 10.2019 for ICD-10-CM codes).  NTDB’s injury definition explicitly excludes the following: late effects of injuries, poisonings, toxic effects, and other external causes; superficial injury; contusion with intact skin surface; and effects of a foreign body entering through orifice.  Our adapted definition further excluded burns (as these are unlikely due to a drug interaction), consistent with work by Sears et al. *J Occup Rehabil* 2015;25(4). |
| Secondary | Typical hip fracture | Closed transcervical fracture (820.00–820.09);  Open transcervical fracture (820.10–820.19);  Closed fracture of unspecified trochanteric section of femur (820.20);  Closed fracture of intertrochanteric section of femur (820.21);  Open fracture of unspecified trochanteric section of femur (820.30);  Open fracture of intertrochanteric section of femur (820.31);  Closed fracture of unspecified part of neck of femur (820.8);  Open fracture of unspecified part of neck of femur (820.9) | Fracture of head and neck of femur (S72.0* with a 7^th^ character modifier of A, B, or C only);  Pertrochanteric fracture (ICD-10-CM S72.1* with a 7^th^ character modifier of A, B, or C only). | Principal inpatient discharge diagnosis on an inpatient hospitalization claim | The inclusion of ICD-9-CM codes for typical closed hip fractures is supported by [FDA Sentinel](https://www.sentinelinitiative.org/sentinel/surveillance-tools/validations-lit-review/health-outcome-algorithm-inventory) and findings by Narogroeknawin et al. *Journal of Clinical Densitometry* 2012;15(1) using 2004–2008 health system data from the University of Alabama at Birmingham. The PPV for typical closed hip fracture ICD-9 discharge diagnosis codes in a principal position was 94%.  ICD-10-CM codes were mapped from ICD-9-CM codes using General Equivalence Mappings. The mapping is partially supported by a validation of Finnish registry data by Sund et al (*Methods Inf Med* 2007;46:558-566) that reported specificities and sensitivities of 99% (95–100%) and 69% (53–84%) for trochanteric fractures and 81% (68–92%) and 97% (92–100%) for femoral neck fractures; these findings were highlighted in a systemic review by Hudson et al (*J Clin Epi* 2013;66:278-285).  The rationale for including typical open hip fractures codes was that such fractures can be caused by an injury.  A hip fracture algorithm including typical open and closed hip fracture codes in 1996–1997 Norwegian data had a PPV and sensitivity of 84% and 90%, respectively (Lofthus et al. *J Clin Epidemiol* 2005;58). Typical open and closed hip fracture codes are a component of [AHRQ’s hip fracture mortality rate quality indicator](https://www.qualityindicators.ahrq.gov/Downloads/Modules/IQI/V50-ICD10/TechSpecs/IQI%2019%20Hip%20Fracture%20Mortality%20Rate.pdf).  The rationale for excluding pathologic fracture codes (e.g., ICD-9 733.14) was that such fractures are due to a localized process such as malignancy or infection (Curtis et al. *Osteoporos Int* 2009;20); their exclusion should have had minimal impact on precision, as ~3% of closed hip fractures are coded as pathologic.  The rationale for excluding atypical (i.e., subtrochanteric and diaphyseal) hip fracture codes (e.g., ICD-9 820.22) was that such fractures are associated with no or minimal trauma and commonly attributed to the use of bisphosphonates and/or glucocorticoids (Shane et al. *JBMR* 2014;29). |
| Secondary | Unintentional motor vehicle crash while individual was driving (MVC) | MVTA, injuring the driver, in collision with train (E810.0);  MVTA, injuring the driver, in re-entrant collision with another motor vehicle (E811.0);  MVTA, injuring the driver, other, in collision with motor vehicle (E812.0);  MVTA, injuring the driver, in collision with other vehicle (E813.0);  MVTA, injuring the driver, in collision with pedestrian (E814.0);  MVTA, injuring the driver, other, in collision on highway (E815.0);  MVTA, injuring the driver, loss of control, without collision on highway (E816.0);  MVTA, injuring the driver, noncollision, while boarding or alighting (E817.0);  MVTA, injuring the driver, other noncollision (E818.0);  MVTA, injuring the driver, unspecified nature (E819.0);  MVNTA, injuring the driver, motor-driven snow vehicle (E820.0);  MVNTA, injuring the driver, off-road motor vehicle (E821.0);  MVNTA, injuring the driver, other, in collision with moving object (E822.0);  MVNTA, injuring the driver, other, in collision with stationary object (E823.0);  MVNTA, injuring the driver, other, while boarding or alighting (E824.0);  MVNTA, injuring the driver, unspecified nature (E825.0) | \| **Identifying motor vehicle accident events of interest using ICD-10-CM code character places** \| \| \| \| \| \| \| \| --- \| --- \| --- \| --- \| --- \| --- \| --- \| \| 1st \| 2nd \| 3rd \| 4th \| 5th \| 6th \| 7th \| \| V \| 2 \| * \| 0 or 4 \| * \| * \| A \| \| V \| 3 \| 0, 1, 2, 3, 4, 5, 6, 7, or 8 \| 0 or 5 \| * \| * \| A \| \| V \| 3 \| 9 \| 0 or 4 \| * \| * \| A \| \| V \| 4 \| 0, 1, 2, 3, 4, 5, 6, 7, or 8 \| 0 or 5 \| * \| * \| A \| \| V \| 4 \| 9 \| 0 or 4 \| * \| * \| A \| \| V \| 5 \| 0, 1, 2, 3, 4, 5, 6, 7, or 8 \| 0 or 5 \| * \| * \| A \| \| V \| 5 \| 9 \| 0 or 4 \| * \| * \| A \| \| V \| 6 \| 0, 1, 2, 3, 4, 5, 6, 7, or 8 \| 0 or 5 \| * \| * \| A \| \| V \| 6 \| 9 \| 0 or 4 \| * \| * \| A \| \| V \| 7 \| 0, 1, 2, 3, 4, 5, 6, 7, or 8 \| 0 or 5 \| * \| * \| A \| \| V \| 7 \| 9 \| 0 or 4 \| * \| * \| A \| \| V \| 8 \| 3, 4, 5, or 6 \| 0 or 5 \| * \| * \| A \| \| Asterisk indicates wildcard \| \| \| \| \| \| \|   MVC events with one of the following any-position emergency department or any-position inpatient discharge diagnoses co-occurring with an ECOI of interest were excluded: intentional self-harm by crashing of motor vehicle (X82*); assault by crashing of motor vehicle (Y03*); or crashing of motor vehicle, undetermined intent (Y32*). | Unintentional traumatic injury (see primary above)  and  Any-position discharge diagnosis, arising from the same hospital presentation as the injury, on an ED or inpatient hospitalization claim | The PPV and sensitivity for the motor vehicle crash component of the outcome definition are 88–89% and 97%, respectively ([LeMier et al. *Inj Prev* 2001;7](https://injuryprevention.bmj.com/content/7/4/334.long) and [Bowman et al. *Perspect Health Inf Manag* 2011;8](https://www.ncbi.nlm.nih.gov/pubmed/22016669)).  The inclusion of motor vehicle accident codes of an unintentional manner and exclusion of self-inflicted, assault, and undetermined manner motor vehicle accident codes is supported by the CDC Injury Center’s [E-Code Grouping Matrix](https://www.cdc.gov/injury/wisqars/ecode_matrix.html). |

**eTable 2.** Characteristics of benzodiazepine and related drug users experiencing a typical hip fracture

|  | | **Benzodiazepine and Related Drugs (N=2,887 persons in total*)** | | | | | | |
| --- | --- | --- | --- | --- | --- | --- | --- | --- |
|  |  | **Alprazolam** | **Clonazepam** | **Diazepam** | **Eszopiclone** | **Lorazepam** | **Temazepam** | **Zolpidem** |
| Persons | Count | 667 | 360 | 171 | 39 | 770 | 176 | 704 |
| Days of observation period, per person | Median  (Q1-Q3) | 67.0  (36.0-214.0) | 86.5  (37.0-229.5) | 37.0  (21.0-116.0) | 63.0  (36.0-142.0) | 48.0  (29.0-138.0) | 62.5 (37.0-155.0) | 60.5 (37.0-122.0) |
| Days of observation | Count | 132,596 | 73,255 | 20,331 | 4,198 | 114,726 | 25,512 | 88,657 |
| Age in years | Median  (Q1-Q3) | 79.1  (71.9-84.5) | 77.3  (68.8-83.3) | 76.5  (67.6-83.6) | 77.9  (70.9-81.7) | 81.1  (74.4-85.2) | 78.9 (73.1-83.9) | 78.5 (70.9-83.2) |
| Sex, count (%) | Female | 529  (79.3) | 279  (77.5) | 133  (77.8) | 30  (76.9) | 596  (77.4) | 126 (71.6) | 485 (68.9) |
| Race, count (%) | White | 500  (75.0) | 269  (74.7) | 132  (77.2) | 26  (66.7) | 572 (74.3) | 120 (68.2) | 543 (77.1) |
|  | African American | 39  (5.8) | 24  (6.7) | 7  (4.1) | 4  (10.3) | 53 (6.9) | 11 (6.3) | 49 (7.0) |
|  | Hispanic | 47  (7.0) | 29  (8.1) | 9  (5.3) | 4  (10.3) | 46 (6.0) | 18 (10.2) | 37 (5.3) |
|  | Asian | 5  (0.7) | 3  (0.8) | 2  (1.2) | 1  (2.6) | 3 (0.4) | 5 (2.8) | 12 (1.7) |
|  | Unknown | 76  (11.4) | 35  (9.7) | 21  (12.3) | 4  (10.3) | 96 (12.5) | 22 (12.5) | 63 (8.9) |

* A person may have contributed to multiple drug episodes in the analysis.

Chlordiazepoxide, clobazam, clorazepate, estazolam, flurazepam, oxazepam, quazepam, ramelteon, suvorexant, tasimelteon, triazolam, and zaleplon were examined but did not have eligible samples with statistically stable models.

**eTable 3.** Characteristics of benzodiazepine and related drug users experiencing a motor vehicle crash while individual was driving

|  | | **Benzodiazepine and Related Drugs (N=629 persons in total*)** | | | | |
| --- | --- | --- | --- | --- | --- | --- |
|  |  | **Alprazolam** | **Clonazepam** | **Diazepam** | **Lorazepam** | **Zolpidem** |
| Persons | Count | 200 | 144 | 67 | 71 | 147 |
| Days of observation period, per person | Median  (Q1-Q3) | 109.0 (37.0-463.5) | 96.0 (37.0-271.0) | 37.0 (21.0-119.0) | 65.0 (37.0-128.0) | 53.0 (37.0-168.0) |
| Days of observation | Count | 64,102 | 35,631 | 11,213 | 13,219 | 24,864 |
| Age in years | Median  (Q1-Q3) | 53.8 (43.9-65.6) | 54.5 (43.2-66.8) | 55.0 (47.6-66.6) | 64.4 (52.6-72.6) | 62.0 (50.3-71.9) |
| Sex, count (%) | Female | 122 (61.0) | 83 (57.6) | 41 (61.2) | 47 (66.2) | 85 (57.8) |
| Race, count (%) | White | 151 (75.5) | 110 (76.4) | 45 (67.2) | 50 (70.4) | 85 (57.8) |
|  | African American | 18 (9.0) | 11 (7.6) | 12 (17.9) | 9 (12.7) | 31 (21.1) |
|  | Hispanic | 9 (4.5) | 9 (6.3) | 4 (6.0) | 4 (5.6) | 10 (6.8) |
|  | Asian | 2 (1.0) | 0 (0.0) | 0 (0.0) | 1 (1.4) | 2 (1.4) |
|  | Unknown | 20 (10.0) | 14 (9.7) | 6 (9.0) | 7 (9.9) | 19 (12.9) |

* A person may have contributed to multiple drug episodes in the analysis.

Chlordiazepoxide, clobazam, clorazepate, estazolam, eszopiclone, flurazepam, oxazepam, quazepam, ramelteon, suvorexant, tasimelteon, temazepam, triazolam, and zaleplon were examined but did not have eligible samples with statistically stable models.

**eTable 4.** Summary data on rate ratios for typical hip fracture, by benzodiazepine and related drugs object drug

|  | **Benzodiazepine and Related Drugs*** | | | | | | |
| --- | --- | --- | --- | --- | --- | --- | --- |
|  | **Alprazolam** | **Clonazepam** | **Diazepam** | **Eszopiclone** | **Lorazepam** | **Temazepam** | **Zolpidem** |
| *Unadjusted analyses* | | | | | | | |
| Number of base pair-candidate interacting precipitant triads examined | 458 | 208 | 37 | No valid models* | 496 | 33 | 327 |
| Range of RRs after semi-Bayes shrinkage, min to max | 0.42-1.16 | 0.54-1.39 | 0.35-0.65 |  | 0.33-0.90 | 0.28-0.51 | 0.40-1.39 |
| Number of triads with statistically significantly elevated ratio of RRs | 0 | 0 | 0 |  | 0 | 0 | 0 |
|  |  |  |  |  |  |  |  |
| *Confounder-adjusted analyses* | | | | | | | |
| Number of base pair-candidate interacting precipitant triads examined | No valid models* | 10 | No valid models* | No valid models* | 241 | No valid models* | 7 |
| Range of RRs after semi-Bayes shrinkage, min to max |  | 0.75-1.07 |  |  | 0.42-1.02 |  | 0.73-0.97 |
| Number of triads with statistically significantly elevated ratio of RRs (i.e., potential 3DI signals) |  | 0 |  |  | 0 |  | 0 |

max = maximum; min = minimum; RR = rate ratio
* Models were run, but none had valid results (i.e., either model did not converge or variance estimate >10)

**eTable 5.** Summary data on rate ratios for motor vehicle crash while individual was driving, by benzodiazepine and related drug

|  | **Benzodiazepine and Related Drugs** | | | | |
| --- | --- | --- | --- | --- | --- |
|  | **Alprazolam** | **Clonazepam** | **Diazepam** | **Lorazepam** | **Zolpidem** |
| *Unadjusted analyses* | | | |  |  |
| Number of base pair-candidate interacting precipitant triads examined | 98 | 65 | 10 | 14 | 42 |
| Range of RRs after semi-Bayes shrinkage, min to max | 0.46-1.08 | 0.50-0.94 | 1.03-1.53 | 1.31-2.27 | 0.62-1.24 |
| Number of triads with statistically significantly elevated ratio of RRs (i.e., potential 3DI signals) | 0 | 0 | 0 | 0 | 0 |
|  |  |  |  |  |  |
| *Confounder-adjusted analyses* | | | |  |  |
| Number of base pair-candidate interacting precipitant triads examined | 12 | No valid models* | No valid models* | No valid models* | No valid models* |
| Range of RRs after semi-Bayes shrinkage, min to max | 0.50-0.79 |  |  |  |  |
| Number of triads with statistically significantly elevated ratio of RRs (i.e., potential 3DI signals) | 0 |  |  |  |  |

max = maximum; min = minimum; RR = rate ratio 
* Models were run, but none had valid results (i.e., either model did not converge or variance estimate >10)

**eFigure 1. Commonly prescribed benzodiazepine and related drug object + co-dispensed drug of the base pair with candidate interacting precipitant associations with unintentional traumatic injury, σ^2^ = 0.25.**

Panels A-E depict associations between 3DIs and unintentional traumatic injury when the object benzodiazepine and related drug (BZD) of interest was alprazolam, clonazepam, lorazepam, zolpidem, and diazepam, respectively. Semi-Bayes shrinkage prespecified a variance of 0.25, assuming that 95% of true rate ratios would fall within an unspecified 7-fold range of each other. The x-axis represents the log base 2 (semi-Bayes shrunk adjusted rate ratio) for BZD + co-dispensed drug of base pair + candidate interacting precipitant vs. BZD + co-dispensed drug of base pair. The y-axis represents the log (1/p-value) for the semi-Bayes shrunk adjusted rate ratio. The value of the X reflects the magnitude of the RR, whereas the value of Y reflects the level of statistical significance. Data points in the upper right quadrant represent statistically significant elevated rate ratios for the association between BZD + co-dispensed drug of base pair + candidate interacting precipitant (vs. BZD + co-dispensed drug of base pair) and injury (i.e., putative 3DI signals). 3DI=drug-drug-drug interactions.


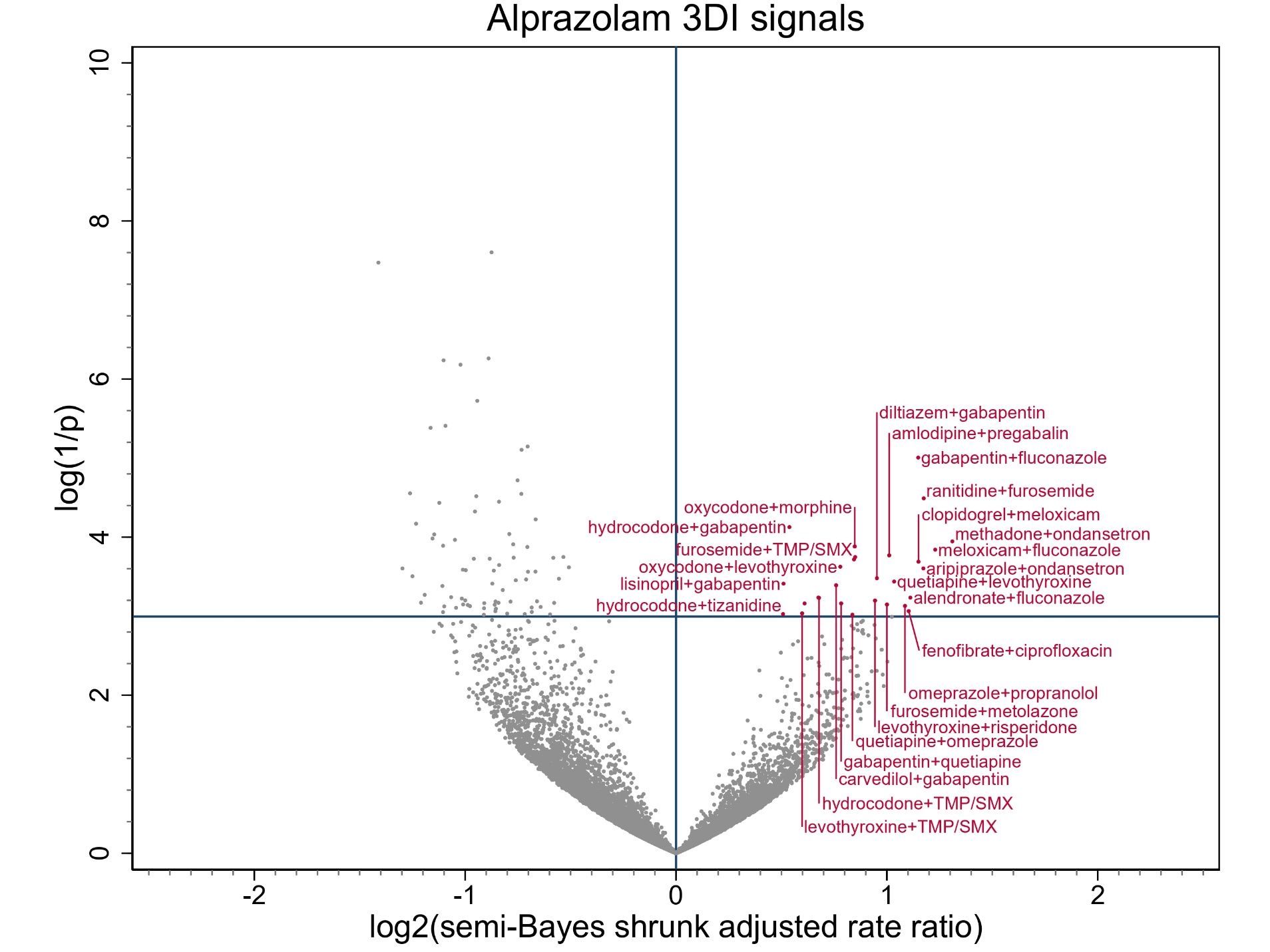

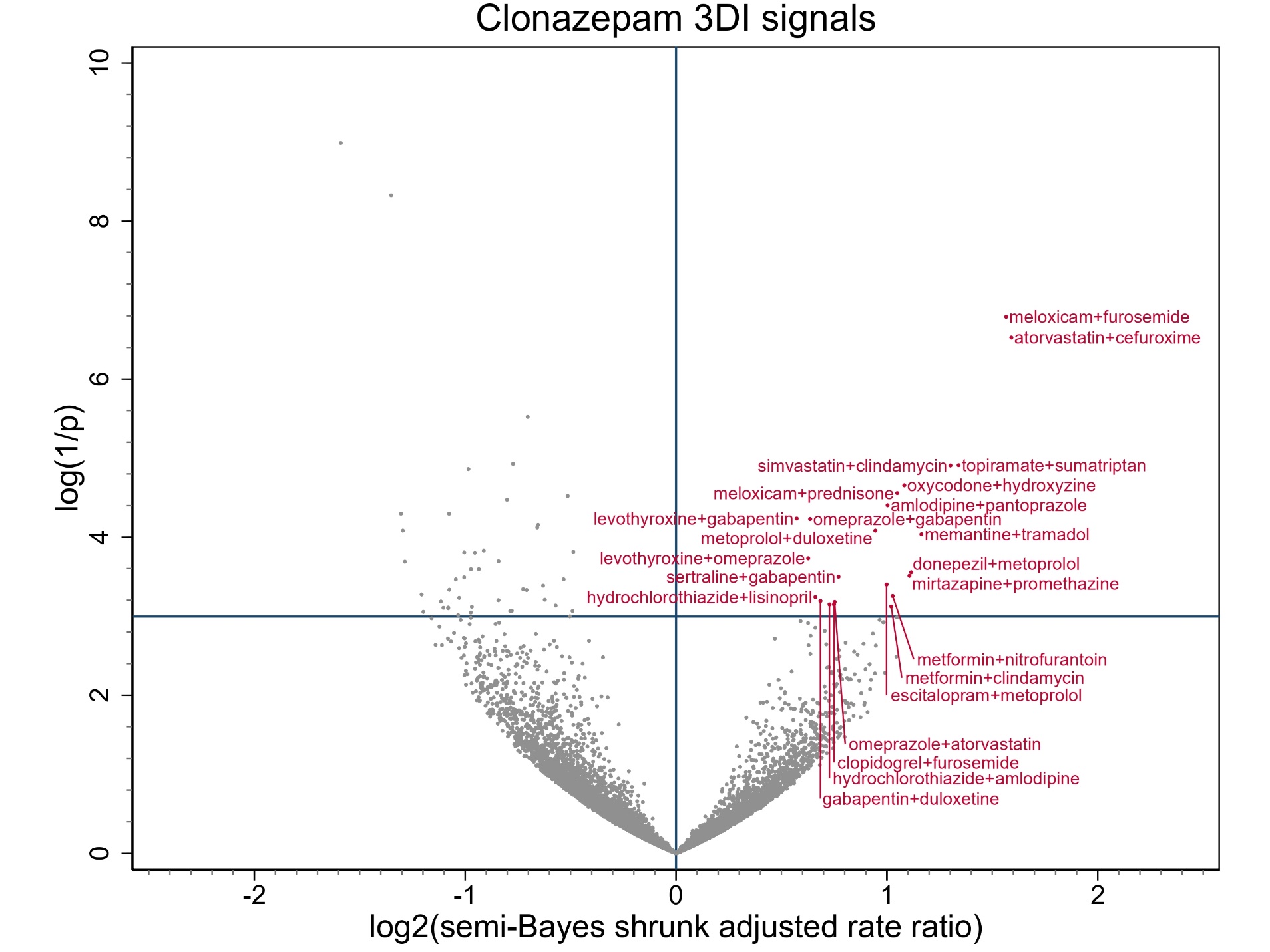

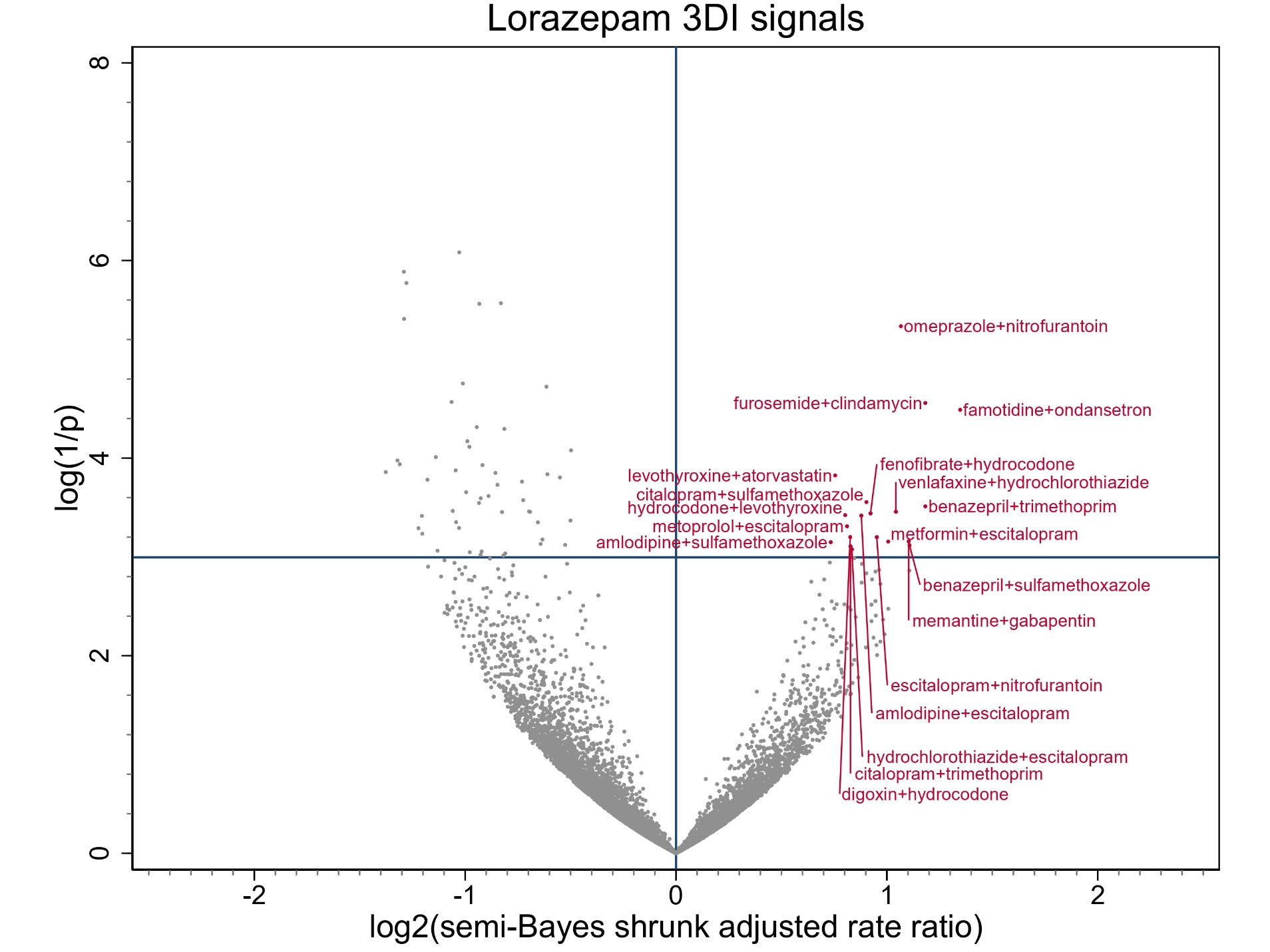

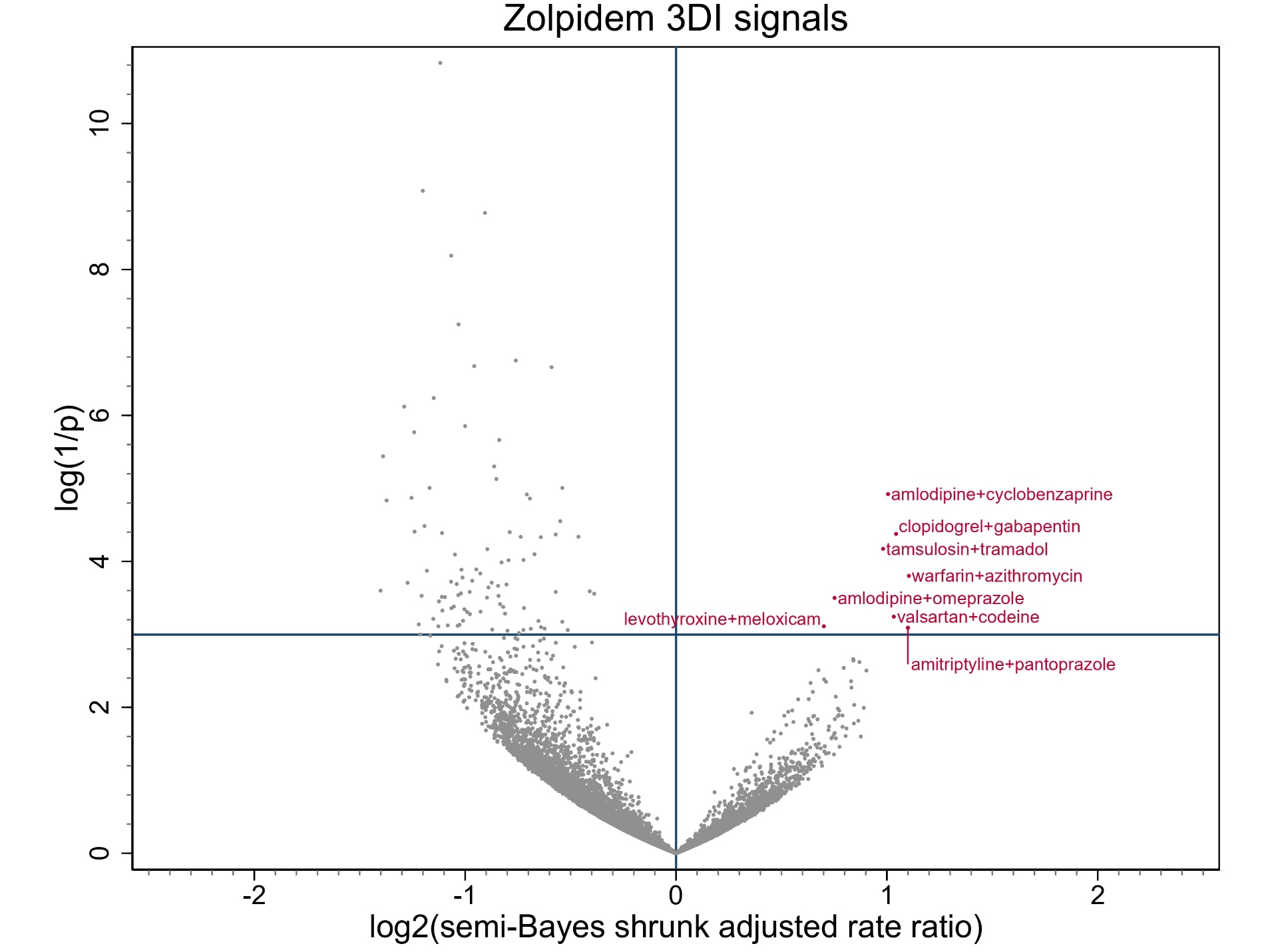

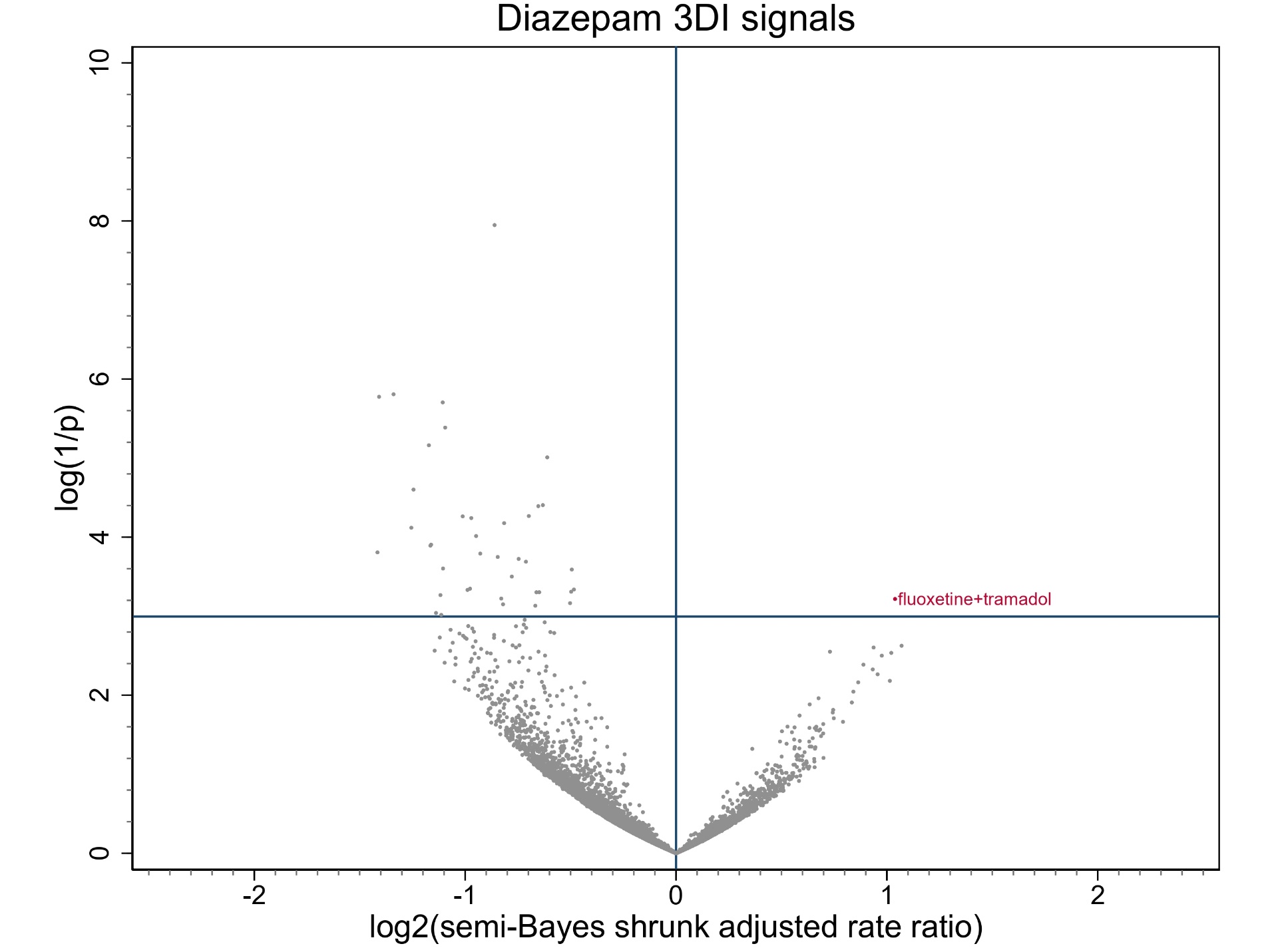


**eFigure1- Panel A**

**eFigure1- Panel B**

**eFigure1- Panel C**

**eFigure1- Panel D**

**eFigure1- Panel E**

**eFigure 2. Commonly prescribed benzodiazepine and related drug object + co-dispensed drug of the base pair with candidate interacting precipitant associations with unintentional traumatic injury, σ^2^ = 0.67.**

Panels A-E depict associations between 3DIs and unintentional traumatic injury when the object benzodiazepine and related drug (BZD) of interest was alprazolam, clonazepam, lorazepam, zolpidem, and diazepam, respectively. Semi-Bayes shrinkage prespecified a variance of 0.67, assuming that 95% of true rate ratios would fall within an unspecified 25-fold range of each other. The x-axis represents the log base 2 (semi-Bayes shrunk adjusted rate ratio) for BZD + co-dispensed drug of base pair + candidate interacting precipitant vs. BZD + co-dispensed drug of base pair. The y-axis represents the log (1/p-value) for the semi-Bayes shrunk adjusted rate ratio. The value of the X reflects the magnitude of the RR, whereas the value of Y reflects the level of statistical significance. Data points in the upper right quadrant represent statistically significant elevated rate ratios for the association between BZD + co-dispensed drug of base pair + candidate interacting precipitant (vs. BZD + co-dispensed drug of base pair) and injury (i.e., putative 3DI signals). For ease of reading, we limited labeling to upper right quadrant data points with log base 2 (semi-Bayes shrunk adjusted rate ratio) ≥1.5 or log (1/p-value) for the semi-Bayes shrunk adjusted rate ratio ≥10. 3DI=drug-drug-drug interactions.


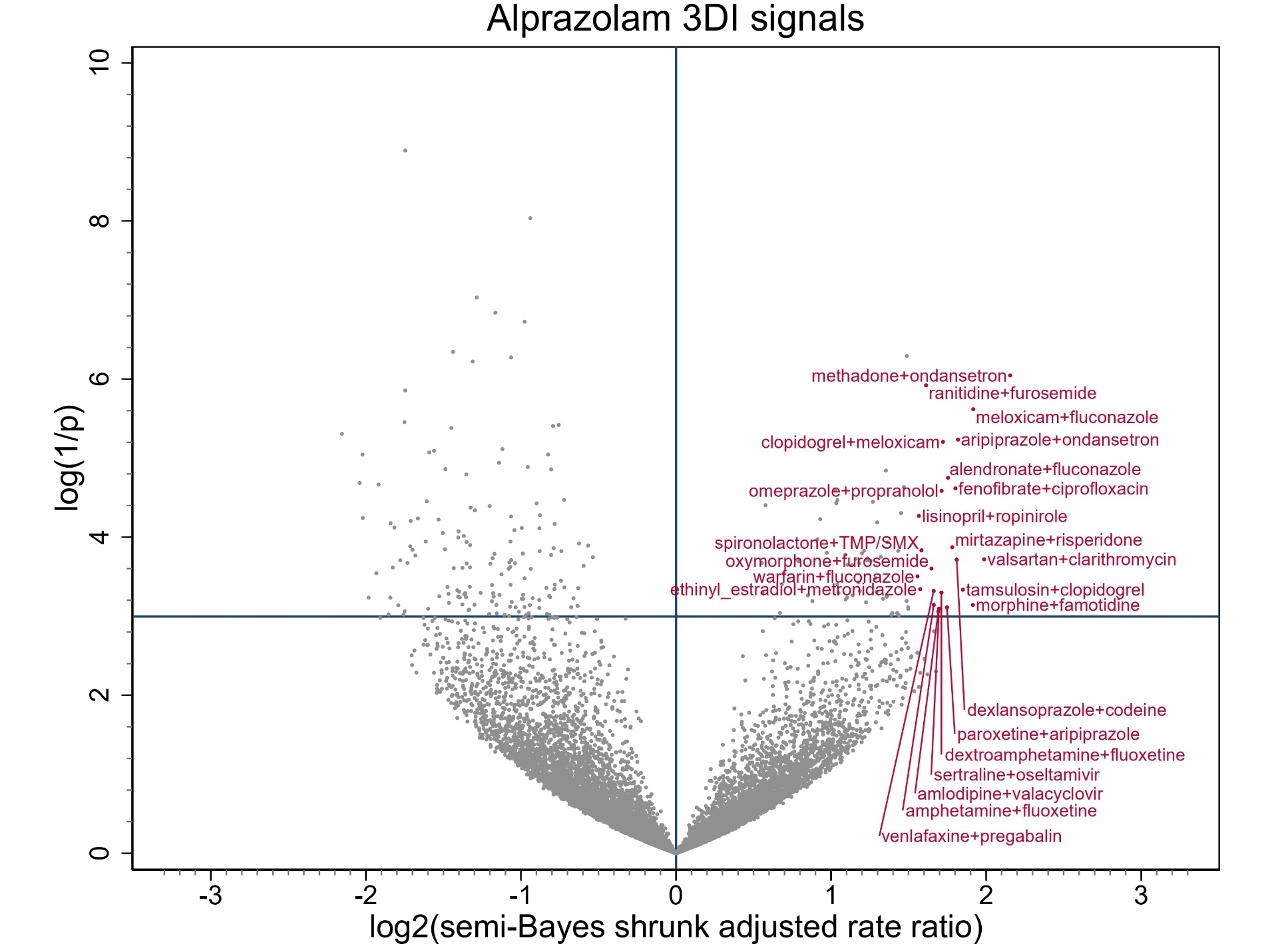

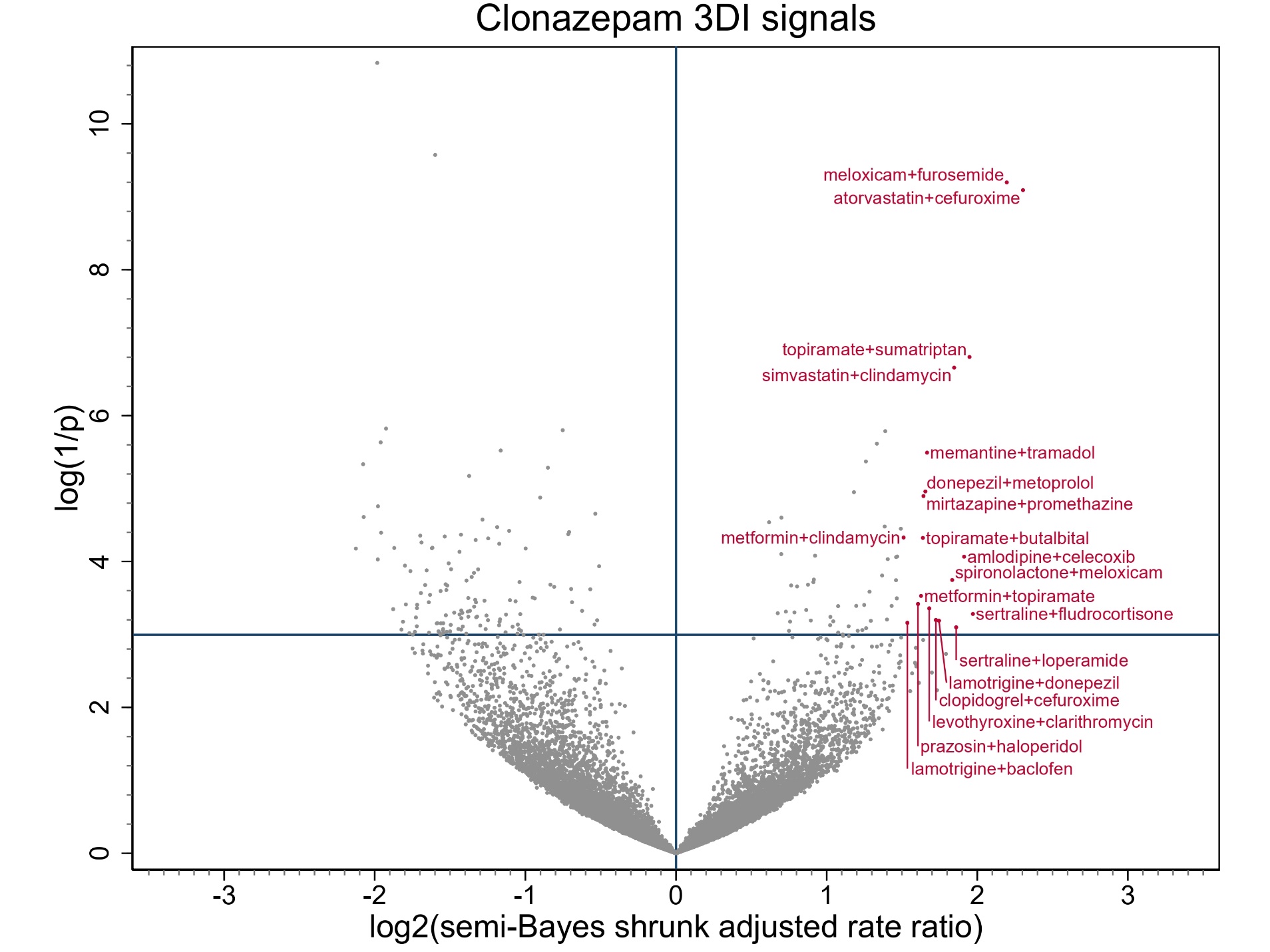


**eFigure2- Panel A**

**eFigure2- Panel B**


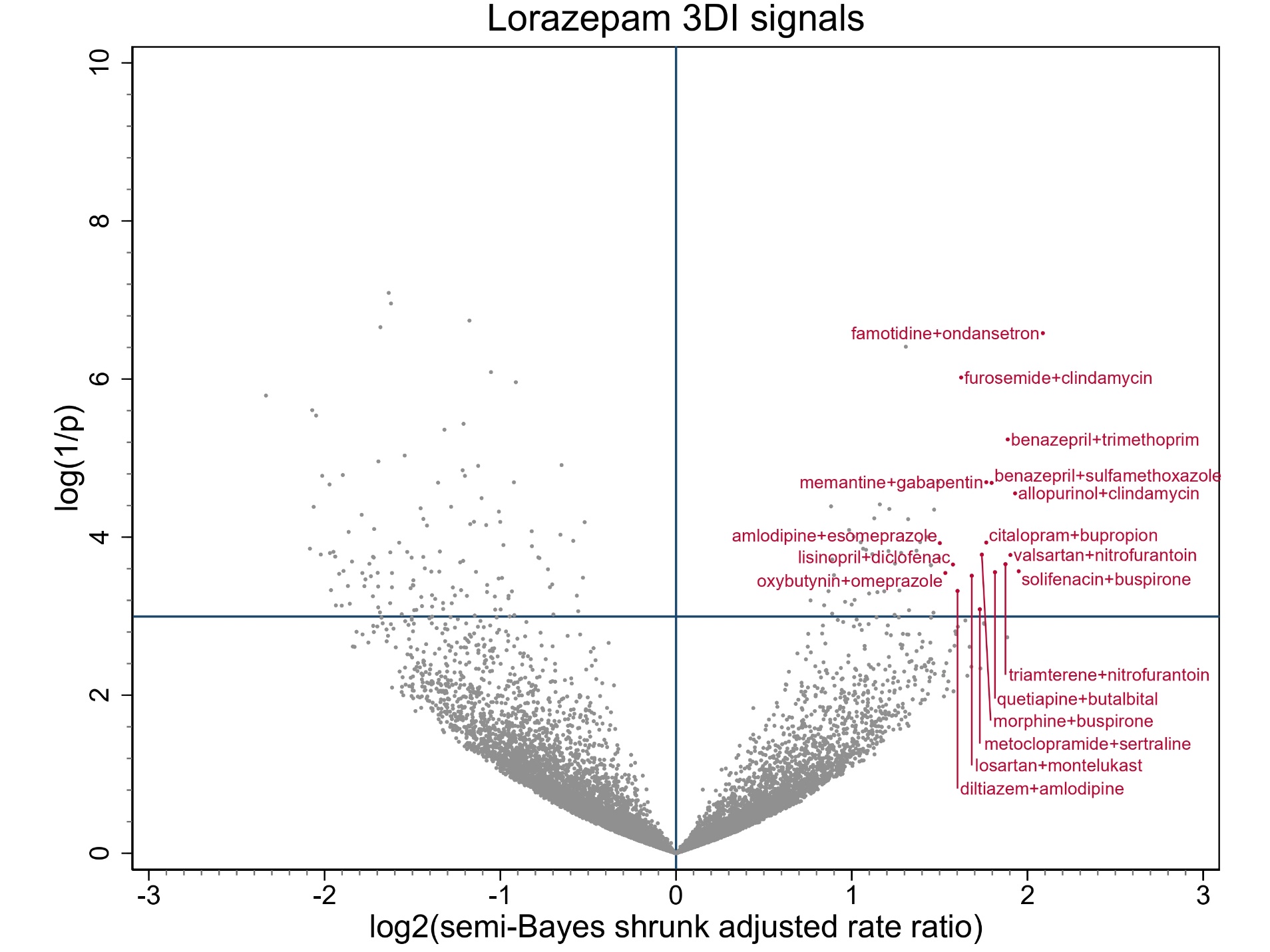


**eFigure2- Panel C**


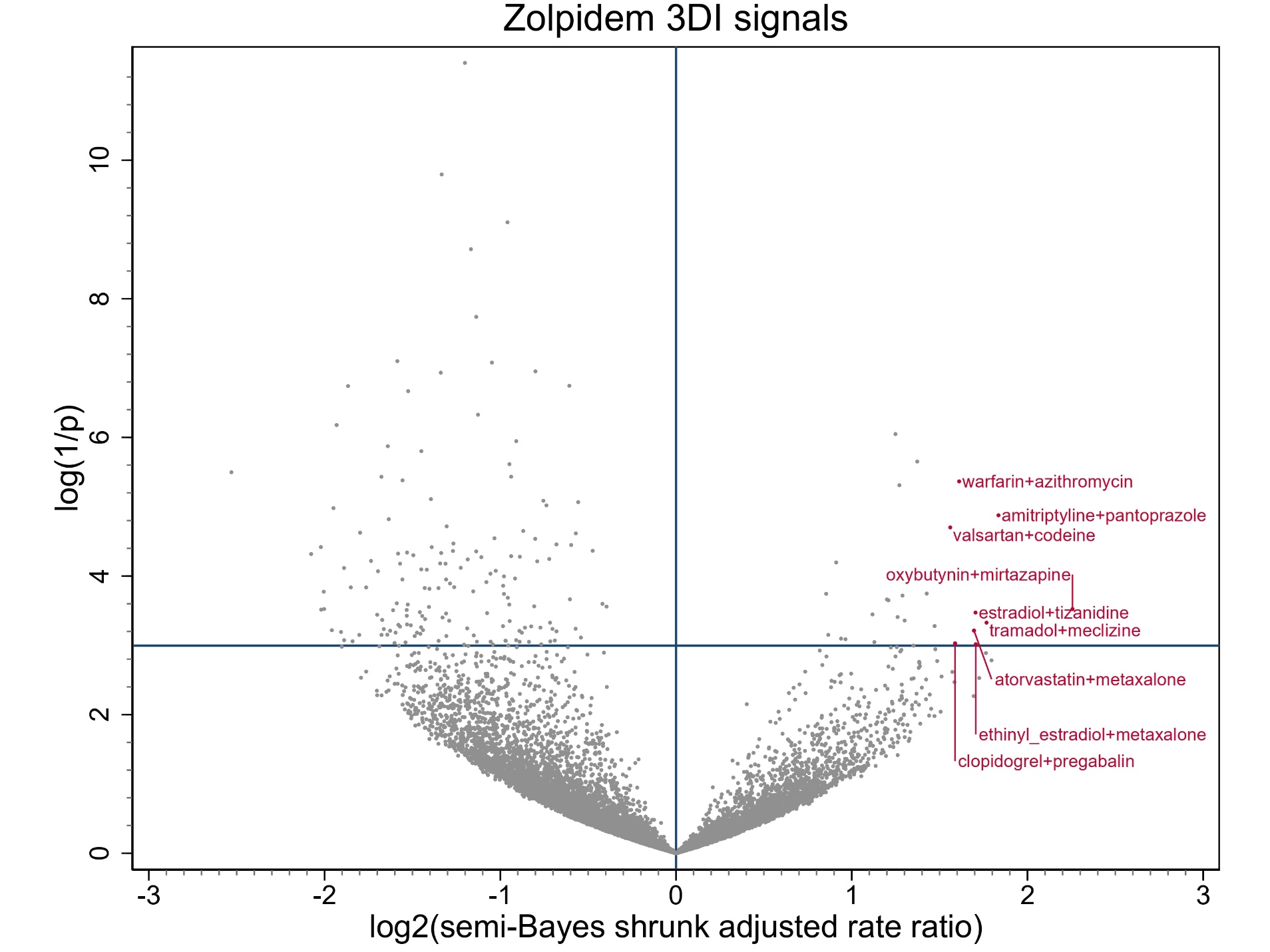


**eFigure2- Panel D**


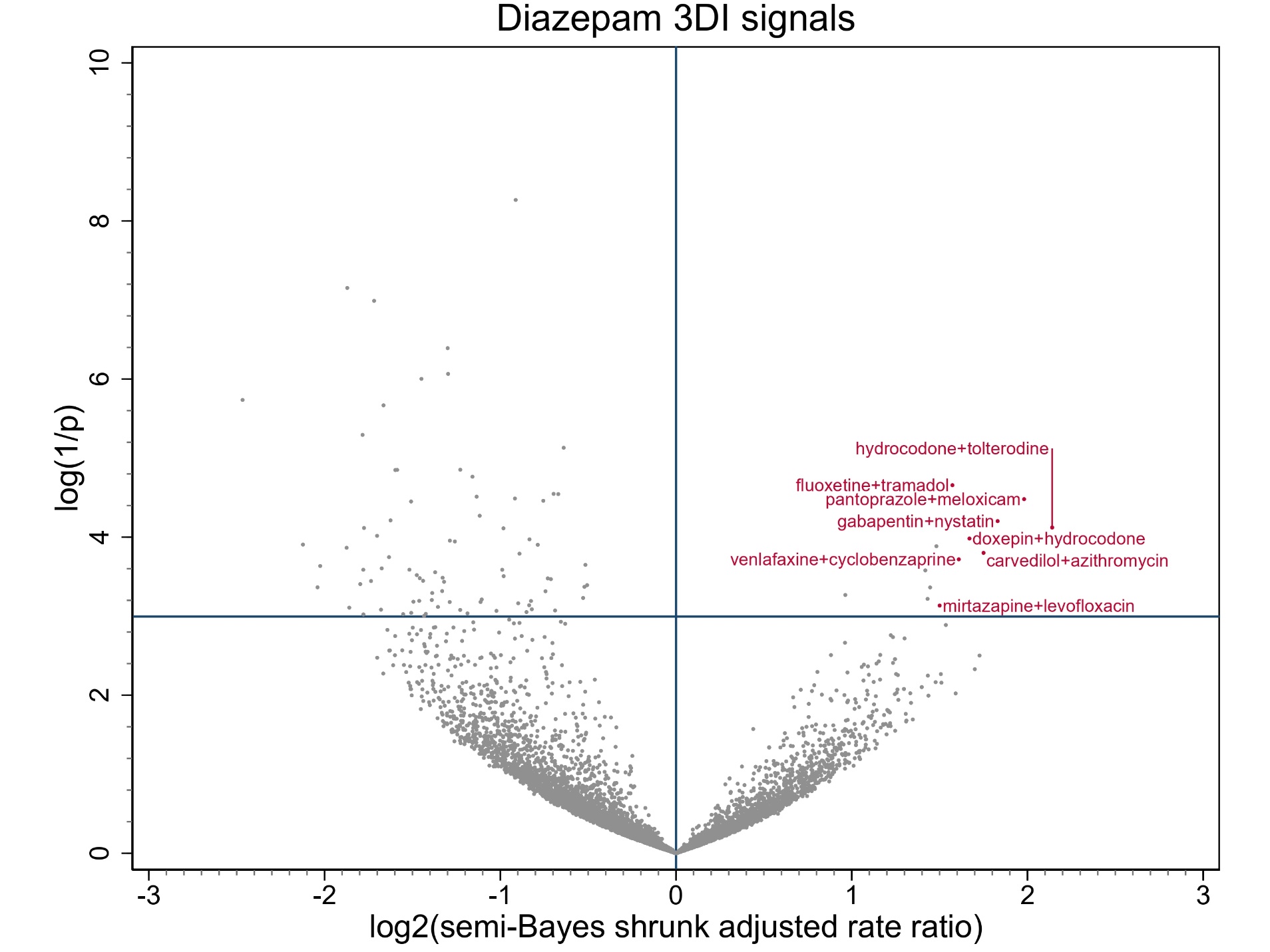


**eFigure2- Panel E**

**eResults.** Links to visualization of all benzodiazepine and related drugs triads screened and their associated rate ratios

- GitHub repo: <https://github.com/wvdmei/3DI-Shiny-Visualization>.
- Link to Shiny app: <https://wvdmei.shinyapps.io/3DI-Visualization/> .
